# Effect of repeated treatment with topiramate on voluntary alcohol intake and beta-endorphin plasma level in Warsaw alcohol high-preferring rats

**DOI:** 10.1007/s00213-012-2812-z

**Published:** 2012-07-31

**Authors:** Jadwiga Zalewska-Kaszubska, Bartosz Bajer, Dorota Gorska, Dariusz Andrzejczak, Wanda Dyr, Przemysław Bieńkowski

**Affiliations:** 1 Department of Pharmacodynamics, Medical University of Lodz, Muszynskiego 1, 90-151 Lodz, Poland; 2 Department of Pharmacology and Physiology of the Nervous System, Institute of Psychiatry and Neurology, Warsaw, Poland

**Keywords:** Alcohol, Beta-endorphin, Warsaw high-preferring rats, Topiramate

## Abstract

**Rationale:**

Pharmacological treatment currently used for alcohol dependence is not sufficient for the all patients, and there is a crucial need to find more effective treatments. Recent studies indicate that topiramate is likely the most promising new medication for alcohol dependence. The rationale for topiramate as treatment for alcohol addiction is based on its multifaceted neurochemical activity that targets multiple neural pathways.

**Objectives:**

This study aims to assess the effect of repeated treatment with topiramate on voluntary alcohol intake and beta-endorphin plasma level in rats selectively bred for high alcohol preference.

**Methods:**

Initially, Warsaw high preferring rats (*N* = 50) were given a 24-h/day free choice between a 10 % (*v*/*v*) alcohol solution and water for three consecutive weeks. Subsequently, rats were administered with topiramate (40 or 80 mg/kg b.w.) or vehicle for 14 days and ethanol intake was measured daily. Subsequently, we examined the effects of topiramate on plasma beta-endorphin levels, while alcohol was available and when it was not available for an extended period time.

**Results:**

We observed significantly increase in the levels of beta-endorphin in rats with free access to alcohol both in a topiramate- or vehicle-treated group. However, in topiramate-treated group, a voluntary consumption of alcohol diminished in comparison with the vehicle-treated rats.

**Conclusion:**

The results from this study indicated that topiramate reduces voluntary alcohol intake and support our previous findings that the increase of beta-endorphin level is responsible at least partly for the effectiveness of drugs in treating the alcohol addiction.

## Introduction

Topiramate, originally developed as an anticonvulsive drug, has been lately discussed as the most promising drug in treatment of alcohol dependence. It has been shown that topiramate effectively prevents different seizures in alcohol withdrawal syndrome in rats (Rustembegovic et al. 2002; Cagetti et al. 2004; Farook et al. 2007). In animal models of alcohol consumption, the drug decreased the intake of ethanol and lowered the preference for alcohol in a free-choice paradigm (Gabriel and Cunningham 2005; Nguyen et al. 2007) but failed to block conditioned place preference to alcohol in mice (Gremel et al. 2006). It has been demonstrated that topiramate attenuates stress-induced increases in alcohol consumption and preference (Farook et al. 2009) and reduces conditioned abstinence behavior in mice (Farook et al. 2007). These findings suggest that topiramate, when applied to dependent animals, may reduce the craving for alcohol. Lynch et al. (2011) also observed that topiramate prevents an increase in ethanol consumption following a deprivation period (i.e., an animal model of relapse).

Clinical trials in alcohol-dependent patients have confirmed the effectiveness of topiramate in comparison with placebo as a means of reducing heavy drinking (Johnson et al. 2004). Some randomized clinical trials have proven that topiramate was effective in reducing the number of days of drinking and increasing the duration of abstinence (Johnson et al. 2004, 2007). Topiramate was significantly better than a placebo in reducing the percentage of heavy drinking days (Johnson et al. 2007). Statistically significant improvements were also seen in the number of abstinence days, the number of drinks per day, and in liver enzyme tests (Johnson et al. 2004, 2007).

Topiramate acts via multiple mechanisms, including modulation of voltage-gated sodium and calcium ion channels (Zona et al. 1997; Angehagen et al. 2004, 2005), blockade of excitatory glutamate neurotransmission through AMPA/kainate receptors (Follett et al. 2004; Gryder and Rogawski 2003), enhancement of gamma-aminobutyric acid (GABA) transmission (White et al. 2000), and inhibition of carbonic anhydrase isoenzymes (Dodgson et al. 2000). These mechanisms may be beneficial when exploited in the treatment of epilepsy. Antagonism to excitatory glutamate neurotransmission at AMPA/kainate receptors and empowering GABA transmission may also be significant in the treatment of alcohol dependence. In treating alcohol dependence, not all of topiramate’s therapeutic mechanisms are yet fully explored. As alcoholism being a multifaceted disease, and for the sake of therapy, one needs medications that act on multiple sites.

Our previous experiments showed that topiramate affects the opioid system, which plays an important role in the development of alcohol dependence. We observed that repeated administration of topiramate significantly increased the beta-endorphin plasma level in WHP rats (Zalewska-Kaszubska et al. 2007). Beta-endorphin is known to play a key role in a mesolimbic reward system; hence, reduced levels of this peptide may be partially responsible for drug craving and physical withdrawal symptoms (Kiefer et al. 2002; Zalewska-Kaszubska and Czarnecka 2005). A correlation between the level of this peptide and alcohol dependence was observed in people with high family risk of alcoholism (Gianoulakis et al. 1989) as well as in alcohol-preferring rats (Zalewska-Kaszubska et al. 2005). Similarly, Kiefer et al. (2006) in his analysis of patients’ median alcohol intake prior to detoxification observed that beta-endorphin plasma concentration in high alcohol preference patients was significantly lower compared with those of low alcohol preference. In our previous studies, we observed that acamprosate and naltrexone, which are presently the most effective drugs in the treatment of alcohol dependence, given chronically increased levels of endogenous beta-endorphin. In the case of acamprosate, these results were confirmed in clinical studies by Kiefer et al. (2006). Subsequently, we examined other drugs in terms of their impact on changes of beta-endorphin level. We have found, for example, that fluoxetine which has only modest efficacy in alcoholism treatment had no effect the level of this peptide (Zalewska-Kaszubska et al. 2008a).

The objective of the present study is to assess the effect of topiramate on voluntary ethanol consumption and to investigate changes in the beta-endorphin plasma level in WHP rats caused by the periodic topiramate administration.

## Materials and methods

### Animals

The experiments were carried out on 50 female adult rats weighing 220–280 g from the *F*_44–45_ generation of the WHP line that were kept under standard laboratory conditions.

The animals were housed individually in stainless steel cages equipped with two graduated drinking tubes, containing tap water or 10 % (*v*/*v*) alcohol. The alcohol solution was prepared from water and a stock solution of 95 % reagent grade ethanol. Following the procedure established by Dyr and Kostowski (2008), the rats had free access to a solution of 10 % (*v*/*v*) ethanol available in the two drinking tubes over the first week, as a sole source of fluid. Food was available ad libitum. This procedure allowed them to become accustomed to drinking from the tubes and to experience the taste and pharmacological properties of alcohol. After the initial period, the rats were given 24 h of free-choice access to 10 % ethanol and water during three consecutive weeks. Alcohol and water intake were recorded, and the tubes were refilled daily. Consumption of ethanol and water was measured daily at the same time, before topiramate or 1 % methylcellulose (vehicle) administration. The position of the alcohol and water tubes was alternated daily to avoid the development of a position preference. Animals were weighed every 3 days. Prior to administration of topiramate or vehicle, the average alcohol and water intakes were calculated for each rat over five consecutive days prior to the day of treatment. The data are expressed as the daily amount of ethanol ingested (g/kg), the total volume of fluid ingested daily (ml/kg) and preference. Alcohol consumption was determined by calculating grams of alcohol consumed per kilogram of body weight. The ethanol preference was calculated by the following formula:

$$ {\text{Ethanol preference }}\left( \% \right) = {{{{\text{intake of ethanol }}\left( {{\text{ml}}\;{\text{k}}{{\text{g}}^{{ - 1}}}\;{\text{da}}{{\text{y}}^{{ - 1}}}} \right)}} \left/ {{{\text{total fluid intake }}\left( {{\text{ml}}\;{\text{k}}{{\text{g}}^{{ - 1}}}\;{\text{da}}{{\text{y}}^{{ - 1}}}} \right) \times {1}00}} \right.}. $$

After the period when baseline ethanol consumption was established, the rats received intragastrically topiramate in doses of 40 or 80 mg/kg body weight, 2 ml/kg b.w., daily or 1 % methylcellulose (1 % MTC) as vehicle over the course of 14 days, according to the schedule in Table 1.

**Table 1:** Experimental groups of rats

Group	Initial procedure	Treatment (14 days)
Control	The control group was given water as its sole source of fluid	1 % methylcellulose as vehicle (1%MCT)
1 % MTC-ethanol	The rats were given free access to 10 % ethanol and water for 21 days	Vehicle during free ethanol access
1 % MTC-water		Vehicle during ethanol withdrawal
TOP40-ethanol		Topiramate (40 mg/kg) during free ethanol access
TOP40-water		Topiramate (40 mg/kg) during ethanol withdrawal
TOP80-ethanol		Topiramate (80 mg/kg) during free ethanol access
TOP80-water		Topiramate (80 mg/kg) during ethanol withdrawal

All experimental procedures were performed in accordance with the Guide for the Care and Use of Laboratory Animals and were approved by the local Animal Research Committee.

### Materials

Sep-pak C18 cartridges were obtained from Waters (MA, USA; catalogue no. WAT 020515). Acetone [high-performance liquid chromatography (HPLC) grade] and trifluoroacetic acid (HPLC grade) were from Baker. Aprotinin was from Finepharm, Sp. z o.o., Poland. Topiramate (Topamax^®^) was purchased from Cilag. Ether was purchased in POCh, Poland. The plasma beta-endorphin radioimmunoassay kit was obtained from Phoenix Pharmaceuticals, Inc., USA.

### Blood sample procedure

Twenty-four hours after the last administration of topiramate or vehicle, the rats were anesthetized with ether and blood samples were collected by heart puncture. Blood samples were collected in tubes containing EDTA (1.6 mg/ml) and gently rocked several times to prevent coagulation. Afterwards, the samples were transferred to centrifuge tubes containing aprotinin (500 KIU/ml) and gently rocked several times to inhibit proteinase activity. The samples were then cooled in an ice-bath. The plasma was separated by centrifugation at 1,600×*g* for 15 min at 4 °C. The plasma was frozen and stored at −20 °C until assessment.

### Solid-phase extraction of peptides from plasma

Beta-endorphin extraction was performed as reported previously (Zalewska-Kaszubska et al. 2007). The procedure was based on use of Sep-pak C18 cartridges.

Before loading on Sep-pak C18 cartridges, plasma in 1-ml volumes was acidified in the same volume of 1 % trifluoroacetic acid (TFA) and centrifuged at 10,000×*g* for 20 min at 4 °C. C18 Sep-columns were activated by passing 2 ml of acetone and then equilibrated twice with 2 ml of 1 % TFA in distilled water. The supernatant of acidified plasma solution was loaded onto the columns. The columns were washed twice with 2 ml of 1 % TFA. Beta-endorphins were eluted with 1.5 ml of 1 % TFA/acetone (25:75) and dried under vacuum. Plasma levels of beta-endorphin were estimated by radioimmunoassay method.

## Statistical analysis

Statistical analysis was performed using analysis of variance (ANOVA) for repeated measures, followed by Newman–Keuls post hoc test. The statistical analyses of data were performed using the Statistica9.0 software program to determine statistical differences between groups. Differences were considered significant when *p* < 0.05. All data are expressed as mean ± SEM.

## Results

**Fig. 1:**
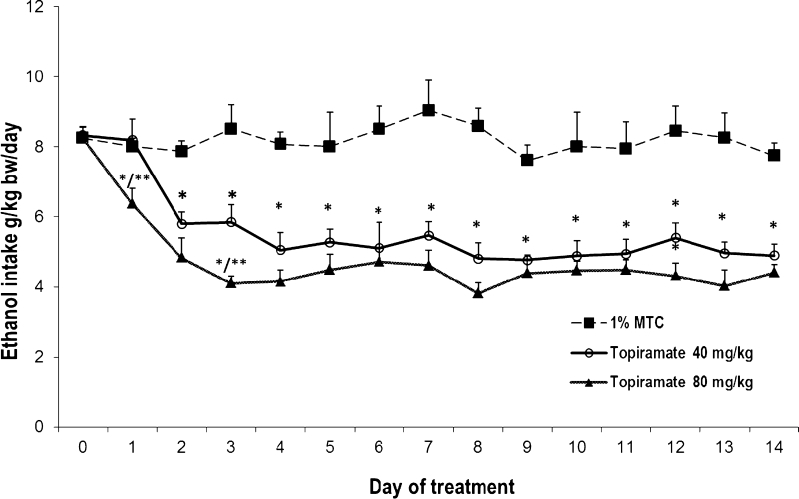
The effect of repeated treatment with topiramate (40 or 80 mg/kg body weight) or 1 % methylcellulose (1%MTC) on ethanol intake by WHP rats given a free choice between ethanol solution (10 % *v*/*v*) and water. Each point represents the mean ± SEM from seven rats. *p < 0.05 in comparison to control rats. **p < 0.05 in comparison to the corresponding group, treated with topiramate in a dose 40 mg/kg

**Fig. 2:**
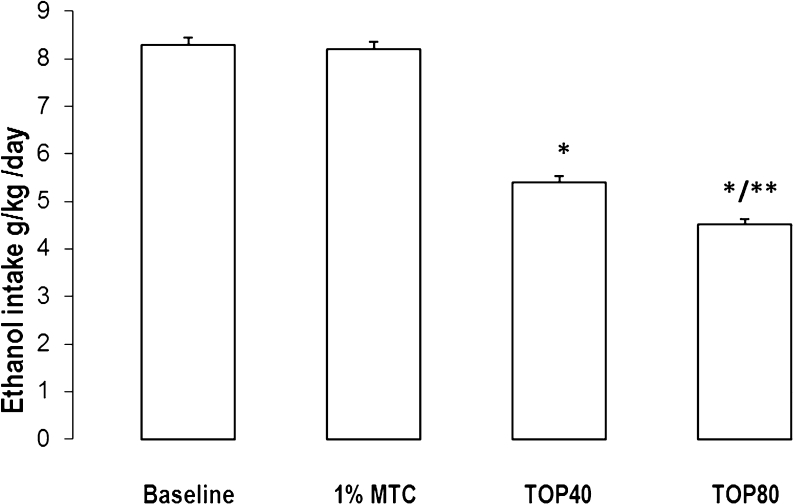
Daily intake of ethanol (g/kg) during the 5-day pretreatment period (baseline) and the 14-day treatment with topiramate 40 or 80 mg/kg body weight (TOP40; TOP80) or 1 % methylcellulose (1%MTC). Rats were given a free choice between ethanol solution (10 % *v*/*v*) and water. Each point represents the mean ± SEM from seven rats. *p < 0.05 in comparison to baseline. **p < 0.05 in comparison to topiramate at dose 40 mg/kg

Analysis of the amount of alcohol consumption in topiramate-treated rats showed a significant decrease in comparison to the baseline. The one-way ANOVA demonstrated a significant effect on mean daily alcohol intake (*F*_5,393_ = 102.19, *p* < 0.001). Baseline voluntary alcohol intake by WHP rats was 8.28 ± 0.17 g kg^−1^ day^−1^ before topiramate treatment. As seen in Fig. 1, repeated administration of topiramate at a dose of 40 or 80 mg/kg for 14 consecutive days significantly reduced voluntary alcohol consumption. Two-way ANOVA revealed treatment effect (*F*_2,252_ = 201.84, *p* < 0.001), day effect (*F*_13,252_ = 2.78, *p* < 0.01), and two-way interaction effect for treatment × day (*F*_26,252_ = 2.03, *p* = 0.30). Overall mean ethanol intake during the topiramate administration period was 5.39 ± 0.14 g kg^−1^ day^−1^ at a dose of 40 mg/kg and 4.52 ± 0.11 at a dose of 80 mg/kg (Fig. 2). Post hoc comparison of two doses of topiramate revealed statistically significant difference in alcohol intake.

As seen in Table 2, administration of topiramate decreased the daily volume of alcohol ingested from 105.1 ± 3.1 ml/kg to 67.5 ± 1.8 ml/kg at a dose 40 mg/kg and from 104.6 ± 3.6 to 57 ± 1.4 ml/kg at a dose 80 mg/kg (*F*_5,393_ =100.73, *p* < 0.001). The total fluid intake declined during the administration of topiramate at both doses (*F*_5,393_ = 10.00 *p* < 0.001). At a dose of 40 mg/kg volume of ethanol, intake was reduced by 35 % and the total fluid intake declined by 13 % during the treatment period. Topiramate at a dose of 80 mg/kg reduced mean ethanol consumption for the 14 days of free choice by 55 % and total fluid intake by 9 %.

**Table 2:** Ethanol, water, and total fluid intake (ml kg^−1^ day^−1^) measured during the 5-day pretreatment period and the 14-day treatment with topiramate or 1 % methylcellulose

Group	Ethanol intake (ml kg^−1^ day^−1^)	Water intake (ml kg^−1^ day^−1^)	Total fluid (ml kg^−1^ day^−1^)	Preference (%)
Pretreatment 1%MCT	104.0 ± 4.1	18.2 ± 1.8	122.2 ± 3.4	84.6 ± 1.5
1 % MTC	103.2 ± 2.2	18.1 ± 1.4	121.2 ± 1.9	85.2 ± 1.1
Pretreatment TOP40	105.1 ± 3.14	18.2 ± 1.4	123.3 ± 2.6	85.0 ± 1.2
TOP40	67.5 ± 1.8*	38.8 ± 1.6*	106.5 ± 2.0*	64.0 ± 1.3*
Pretreatment TOP80	104.6 ± 3.6	17.5 ± 2.1	122.0 ± 2.7	85.4 ± 1.7
TOP80	57.3 ± 1.4*^,^**	54.2 ± 2.0*^,^**	111.4 ± 2.0*	52.1 ± 1.3*^,^**

Data are expressed as the means ± SEM from seven rats in each animal group over test sequences: baseline drinking period (5 days) and during topiramate (TOP40—40 mg/kg; TOP80—80 mg/kg body weight daily) or 1 % methylcellulose (1 % MTC) treatment (14 days)

*p < 0.05 in comparison to pretreatment and 1 % methylcellulose treatment groups

**p < 0.05 in comparison to group treated with topiramate in dose of 40 mg/kg

The preference for ethanol changed from 85 to 64 % and 52 % at doses 40 and 80 mg/kg, respectively (*F*_5,393_ = 123.65, *p* < 0.001).

**Fig. 3:**
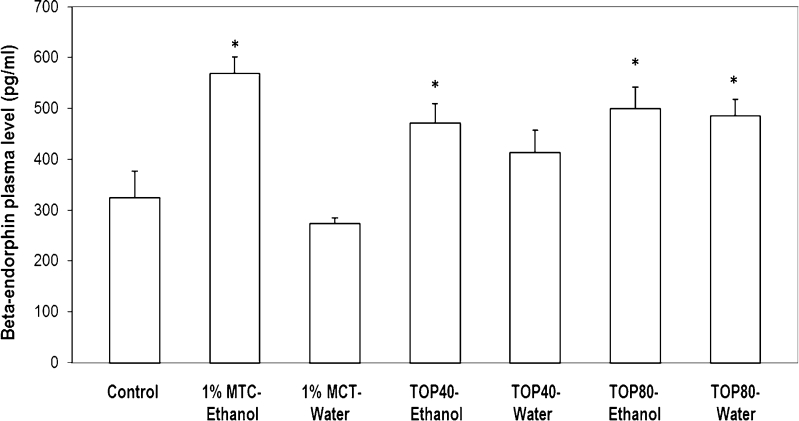
The effect of repeated treatment of topiramate on beta-endorphin plasma levels in WHP rats. Rats were treated intragastrically with topiramate (40 or 80 mg/kg b.w.) or 1 % methylcellulose (1%MTC) for 14 days. Experimental groups: control—group treated with 1 % methylcellulose; 1%MTC-ethanol—group treated with 1 % methylcellulose, with free access to ethanol and water; 1%MTC-water—alcohol withdrawal treated with 1 % methylcellulose; TOP40-ethanol and TOP80-ethanol—groups treated with topiramate in dose of 40 or 80 mg/kg for 14 days with free access to ethanol; TOP40-water and TOP80-water—groups treated with topiramate in dose of 40 or 80 mg/kg for 14 days without access to ethanol. Except for the alcohol-free control group, remaining groups were given free access to 10 % *v*/*v* ethanol during the 3 weeks before experiment. Values are expressed as mean ± SEM from seven to eight rats per group. *p < 0.05 in comparison to control group

The one-way ANOVA demonstrated a significant main effect for beta-endorphin concentration (*F*_6,43_ = 7.50, *p* < 0.001). As shown in Fig. 3, the basic plasma beta-endorphin level in WHP naive rats was 324 ± 52 pg/ml. In the group of rats with free access to alcohol, the levels of this peptide increased to 569 ± 32 pg/ml. Levels of beta-endorphin observed in rats treated with topiramate at doses of 40 and 80 mg/kg concomitant with ethanol access were 473 ± 38 and 485 ± 33 pg/ml, respectively. In withdrawal period, the level of beta-endorphin was 432 ± 44 at dose 40 mg/kg (*p* = 0.051) and 485 ± 33 at dose 80 mg/kg (*p* = 0.005) in comparison to control group. No significant differences were observed in rats treated with topiramate during alcohol withdrawal in comparison to the corresponding group with free access to ethanol. With the exception of TOP40-water group, beta endorphin level in all other groups treated with topiramate was not statistically different from the untreated group with the free alcohol (1%MCT-ethanol). Plasma beta-endorphin levels after a period of 14 days of enforced abstinence from ethanol consumption was 274 ± 11 pg/ml (*p* = 0.36).

## Discussion

The present study indicates that topiramate reduces consumption and preference of alcohol in WHP rats. Similar findings have been reported by other research group that used different strains of rats and mice (Gabriel and Cunningham 2005; Breslin et al. 2010). Studies by Hargreaves and McGregor (2007) have found that topiramate (in doses of 40 and 80 mg/kg) tended to transiently reduce alcohol consumption under ad libitum access. Such reduction of consumption ceased with repeated (7-day) administration of the drug.

In our study, based on the use of topiramate in doses of 40 and 80 mg/kg b.w. for the period of 2 weeks, we did not observe any development of tolerance to the suppressant effect of the topiramate on alcohol intake. Decreased alcohol consumption was accompanied by significant increase in water intake in both the 40 and 80 mg/kg doses of topiramate treated groups. However, in contrast to the studies by Hargreaves and McGregor (2007) and by Gabriel and Cunningham (2005), we observed a reduced overall consumption of all fluids. The mechanism by which topiramate reduces total fluid intake is unclear. This noticeable decrease in total fluid intake complicates our conclusion with regard to the effect of topiramate on ethanol intake as topiramate reduces appetitive behavior, for all fluids. It is probable that topiramate disrupts the regulation of the ingestive process.

Breslin et al. (2010) examined the effects of acutely administered topiramate on ethanol consumption in ethanol-preferring (P) rats and their background strain Wistar in order to assess the relationship between the level of consumption and a treatment effect. They observed that topiramate treatment produced a modest, but persistent (average of 5 days), reduction in ethanol consumption only in alcohol preferring rats. Conversely, in Wistar rats, topiramate had no effect on ethanol consumption or preference. They concluded that effectiveness of topiramate may depend on genetic vulnerability but not on the level of drinking.

This is consistent with our previous study where we observed that the administration of topiramate may have different effects on the opioid system depending on the individual genetic susceptibility to alcoholism (Zalewska-Kaszubska et al. 2007). In alcohol preferring rats (WHP), which differ in the basic plasma beta endorphin concentration from non-preferring rats (WLP), a single application of ethanol resulted in a smaller increase in beta-endorphin in the rats previously treated with topiramate as compared with control rats treated with vehicle. By contrast, in WLP rats, topiramate treatment did not influence the ethanol effect. It has been suggested that topiramate has a modulating effect on the opioid system depending on genetic vulnerability. We agree with Breslin et al. (2010) that the genetic vulnerability may be essential to understanding topiramate’s efficacy in decreasing alcohol intake. That opinion that topiramate may have a different impact based on genetic predisposition to alcoholism opens up a possibility of optimizing the treatment of alcoholics. Our study provides only preliminary suggestions regarding the efficacy of topiramate. Further studies are required to confirm our assumptions and correct our omissions. Thus, the subsequent research should concentrate on other lines of preferring alcohol rats as well as on clinical trials in patients with genetically determined susceptibility to alcohol abuse.

Among major challenges in the treatment of alcoholism are the diminution of physical withdrawal syndrome and craving, and the prevention of relapse into heavy drinking. As some studies suggest, a low level of endorphins may be related the increased craving for alcohol (Van Ree 1996; Kiefer and Wiedemann 2004), as well as to the tendency for alcohol abuse (Zalewska-Kaszubska and Czarnecka 2005). Although we observed differences in reduction of alcohol consumption between the two studied doses of the drug, our observation that topiramate increases the beta-endorphin levels leads us to conclude that even low dose of the drug may be effectively used in the treatment of alcohol dependence. The overall decrease in the alcohol intake at a dose of 80 mg/kg was significantly lower; however, that noticeable difference in the drug’s effectiveness was observed only on first and third day of topiramate application. There was no significant difference in the effect on the beta-endorphin concentration between the two doses of topiramate, as a dose 40 mg/kg was at the limit of significance in comparison to the control group (*p* = 0.051).

The results obtained in this study may support conclusion from our previous research that the effectiveness of treatment of alcoholics may be in part dependent on the means available to increase the beta-endorphin level. We agree with the study by Chiu et al. (2007), which suggested that even a low dose of topiramate can be beneficial in preventing alcohol relapse as even lower doses of the drug partially increased the beta-endorphin level. Miranda et al. (2008) studied effects of topiramate in target doses of 200 and 300 mg/day that were administered to patients within a 32-day titration period to study the stimulating effects of alcohol ingestion. In this laboratory human study, topiramate did not show any effect on the frequency of drinking; however, as a result of its application, subject patients had significantly lower amount of alcohol intake during their drinking episodes as compared to those treated with placebo. On the basis of observation during the titration period, the authors suggested that doses of topiramate as low as 100–175 mg may be effective in reducing heavy drinking. They also observed that significant effect of the drug occurred as early as in the second week of the treatment at the level of only 75 mg a dose, in subjects treated with target dose of 200 mg. However, no significant effect was observed in group receiving 300 mg. Miranda et al. (2008) concluded that the lowering of alcohol intake caused by topiramate is not dependent on the attenuation craving for alcohol. The authors suggested that topiramate may exert its beneficial effects by altering the subjective experiences of alcohol consumption. In addition, Paparrigopoulos et al. (2011) suggested that low-dose topiramate as an adjunct to psychotherapeutic treatment is effective in reducing alcohol craving, as well as in reducing the symptoms of depression and anxiety observed during the early phase of alcohol withdrawal.

Several trials revealed that topiramate is more efficacious than naltrexone. One study of topiramate versus naltrexone in the treatment of alcohol dependence lead to a conclusion that both medicines were effective; however, topiramate was superior to naltrexone on critical measures of drinking, although the study did not have adequate statistical power to establish this fact (Flórez et al. 2008). Suggestive evidence for superiority of topiramate over naltrexone was obtained by Baltieri et al. (2008). Last clinical trial has shown that topiramate at a mean dose of 200 mg/day was better than naltrexone at a usually used dose of 50 mg/day at reducing alcohol intake and cravings throughout a 6-month evaluation (Flórez et al. 2011). Previously, we observed that naltrexone after repeated treatment increased the beta-endorphin level with concomitant reduction in alcohol consumption (Zalewska-Kaszubska et al. 2008b). While topiramate also increases levels of beta-endorphin, its mechanism of action is multidirectional and targets multiple neuronal pathways. Alcohol dependence involves adaptive changes in various neurotransmitters, which makes continuation of abstinence difficult. Due to its versatile action, topiramate may be more effective than naltrexone. In contrast, fluoxetine that does not influence the beta-endorphin level has only limited value in the treatment of alcohol dependence (Zalewska-Kaszubska et al. 2008a).

In summary, the current study supports previous findings that topiramate reduces alcohol consumption, and that the mechanisms underlying this effect may involve, at least in part, modulation of the opioid system. Since all currently used drugs present only moderate effectiveness, this study allow us to look more optimistically into the future of alcohol addiction treatment.
